# Secondary breast cancer after Hodgkin lymphoma: a case report and literature review

**DOI:** 10.3332/ecancer.2018.810

**Published:** 2018-02-14

**Authors:** Joaira Bakkach, Mohamed Mansouri, Ali Loudiyi, Naima Ghailani Nourouti, Amina Barakat, Mohcine Bennani Mechita

**Affiliations:** 1 Biomedical Genomics and Oncogenetics Research Laboratory, Faculty of Sciences and Techniques of Tangier, University Abdelmalek Essaâdi, Tangier 416, Morocco; 2 Oncology Clinic AL AMAL of Tangier, 90 060, Morocco

**Keywords:** Hodgkin lymphoma, secondary breast cancer, radiotherapy, cancer survivorship

## Abstract

The occurrence of secondary breast cancers in women previously exposed to chest irradiation for Hodgkin lymphoma (HL) is considered as a major issue for the quality of life of these long-term survivors as well as a challenge for clinical management. This study reports a case of a woman treated for HL at the age of 24 years, who developed breast cancer after an interval of 20 years. This case highlights once again the importance of awareness among HL survivors about their increased breast cancer risk and re-launches the debate about the efficacy of adoption of breast screening guidelines.

## Background

Patients treated for Hodgkin lymphoma (HL) are faced with several late complications including the occurrence of secondary cancers, which is still a challenging issue in clinical oncology [1]. Breast cancer is the most common solid tumour in female survivors with an estimated relative risk of 8.23 compared to the general population [2]. Based on this increased risk, several international recommendations have been established for breast surveillance [3–5], but published data on surveillance practices raise concerns [6–8]. Up to 87% of HL survivors do not join adequate breast screening programmes [7], and almost half of them seem to be unaware of their increased risk [6].

This study presents a case report of a woman, who was treated for HL at the age of 24 years and developed breast cancer after an interval of 20 years. We also critically discuss current preventive strategies, surveillance, and treatment modalities in secondary breast cancers with a focus on current guidelines.

## Case report

A 44-year-old premenopausal woman without family history of breast cancer was diagnosed with ulcerous-budding bleeding breast tumour at the Oncology Clinic AL AMAL of Tangier (Morocco) with an 18-month history. On clinical examination, she was found to have a bleeding ulcerous mass in the upper outer quadrant of the right breast without axillary adenopathy (T4dN0Mx).

She had been diagnosed with HL Classical Nodular Sclerosis subtype at the age of 24 years (1998). There was no evidence of bone marrow involvement on bone marrow biopsy. The disease was staged III according to Ann Arbor classification. The patient was treated by three cycles of MOPP chemotherapy (mechlorethamine, vincristine, procarbazine and prednisone), followed by 36Gy supra and infradiaphragmatic radiotherapy with up to 50Gy localised boost on the left latero-cervical chain.

Infiltrating ductal carcinoma of the right breast, grade 2 was confirmed by biopsy. Estrogen receptors were positive in 70% of tumour cells, progesterone in 60% and Human Epidermal growth factor Receptor 2 (HER2) was scored 1+. Chest–abdomen–pelvis computed tomography revealed totally or partially calcified or hypodense masses, and nodules within mediastinum, right hilum and abdomen that were attributed to her previous HL. No bone metastases were found on the bone scan.

The patient was offered eight cycles of neoadjuvant chemotherapy based on adriamycin/cyclophosphamide and Taxotere (4 AC60 and 4 docetaxel), with partial clinical response (70%). She subsequently underwent right mastectomy with level II axillary lymph node dissection. Histopathology examination showed a residual tumour classified pT3N2M0, grade 2. Following mastectomy, she received chest wall radiotherapy with a total dose of 50Gy given at 2Gy per fraction in 25 sessions over 5 weeks. Lymph nodes were not irradiated given her prior history of radiotherapy for HL. Radiation therapy was performed without significant complications, except a moderate radiodermatitis (grade 2) for which a local treatment was given. The patient received hormone therapy with Tamoxifen.

In April 2015, she presented with a skin nodule within the mastectomy scar. Cutaneous excision revealed locally recurrent infiltrating ductal carcinoma, grade 2, without endo-vascular emboli. In January 2017, she re-presented with an indurated left axilla associated with small satellite lymphadenopathy. Biopsy of the induration indicated fibrous mastosis without signs of malignancy. Similarly, axillary lymph node biopsy showed no suspicious malignant cells.

## Discussion

It is well established that HL survivors have an increased risk of developing breast cancer when compared to the general population [1, 2, 9–14]. The cumulative risk has been shown to be almost similar to that of BRCA1 mutation carriers and to be 3-fold higher as compared with BRCA2 carriers (35% versus 31% and 10%, respectively) [9]. Importantly, this risk seems to start increasing 5–9 years following the treatment, reaches a peak after 15–19 years [2] and it persists even up to 40 years of follow-up [1, 12].

The risk of secondary breast cancer occurrence was shown to be most influenced by age at treatment and importantly time since treatment and dose (>40Gy yielding greatest risk) [2]. This risk seems to be higher among women exposed early to irradiation with the highest relative risk of 68.7 at 15 years or younger, and to be not significant among women diagnosed after the age of 40 [2] or 50 years [10]. Of note, our patient was diagnosed and treated at the age of 24 years (1998).

Treatment type, most notably radiotherapy with extensive field has been shown as an important risk factor for developing second breast cancer. In a study by De Bruin *et al*. [13], mantle irradiation has been associated with 2.7-fold increased relative risk compared to mediastinal irradiation alone. Furthermore, combination of any chemotherapy appears to promote the radiotherapy effect, and this was even more pronounced if chemotherapy was based on alkylating agents such as mechlorethamine and procarbazine [2]. However, other studies have demonstrated that additional alkylating chemotherapy is associated with reduced risk, and this was attributed to the protector effect of early menopause induced by this gonadotoxic chemotherapy [11–13]. In a cohort study published by Swerdlow *et al*. [12], individualised risk estimations have been provided according to several factors. For instance, a patient, who has been treated 20 years previously with ≥40Gy mantle radiotherapy combined to alkylating chemotherapy at the age of 20–35 years, had a cumulative risk of 10.6 in the next 10 years. Our patient received MOPP chemotherapy followed by supra and infradiaphragmatic radiotherapies with a total cumulated dose of 86Gy, consistent with the guidelines available in the era when she was treated (1998).

Secondary breast cancers following HL generally occur at least 20 years younger than that of sporadic cancers with a median age of approximately 40 years [11, 14, 15]. Our patient was 42 years old at diagnosis.

These tumours were reported to be often bilateral [15], occur more frequently in the margins of radiotherapy field [2, 14], and to be more likely located on external quadrants [10].

Cardiac irradiation and anthracycline-based chemotherapy received during treatment for HL increase the risk of cardiac complications with an estimated 20-year cumulative incidence rate of 16% [16].

This increased risk of cardiotoxicity may unfortunately limit the therapeutic options for women, who develop secondary breast cancer. Thus, several important clinical questions are raised. For instance, is there a place for conservative therapy for breast cancer patients with tumours in the left breast, or should they be treated systematically by mastectomy in order to avoid radiotherapy that may increase their cardiotoxicity risk? Is there a place for chest wall radiotherapy post mastectomy for the poor-prognosis breast cancer group with tumours in the left breast? Another raised issue is related to HER2-positive breast cancers - what will be the appropriate duration for Trastuzumab in this setting in light of the increased risk of cardiotoxicity with previous anthracycline and radiotherapy treatment? As these questions seem to be unresolved in randomised trials (including HL patients), individualised decision making by a multidisciplinary team remains the best option for second breast cancer management.

Mastectomy has long been described as the preferred surgical option in order to avoid further sequelae related to re-irradiation after conserving surgery, such as cardiotoxicity as well as tissue necrosis, which could develop many years thereafter [14]. However, the role of conservative therapy has been re-evaluated in some studies that have reported satisfying locoregional control and acceptable cosmetic results, suggesting that it could be a possible option for these survivors [17–30] (Table 1). Interestingly, despite the limited number of recruited patients and the low evidence level, some of these reports have shed light on promising radiotherapy techniques that could reduce mastectomy numbers including the intraoperative radiotherapy with electrons (ELIOT), partial breast irradiation (PBI) by brachytherapy or three-dimensional (3D) conformal techniques. There is a great need for prospective studies to validate these therapeutic options in HL survivors, and to determine the groups of patients who could benefit from it.

A Phase II trial conducted by the Radiation Therapy Oncology Group is underway in order to evaluate adverse events after 3D conformal PBI in locally recurrent breast cancer patients treated by repeat breast conserving surgery (NCT01082211) [31]. As clinical trials involving survivors with secondary breast cancer after HL are lacking, extrapolation from such studies could be important and may expand the treatment options for this group of patients.

Our patient underwent mastectomy due to the advanced stage of her disease (T4dN0M0). Based on the radiation oncologists’ view and considering her good skin status, she was offered subsequent radiotherapy in the right chest wall, but without lymph nodes irradiation because of her prior irradiation for HL.

Over the years, with the accumulating evidence supporting the increased risk of late complications related to older therapy modalities, the latter have been adapted and evolved significantly. Extended-field irradiation (mantle-type, lumbosplenic bar or inverted-Y), which was the standard of care for many decades has been replaced by involved field radiotherapy (IFRT). Based on this evolutionary concept of IFRT, several techniques have been developed later such as involved node radiotherapy and involved site radiotherapy [32]. The higher doses routinely administered in the past were also de-escalated from more than 40Gy to 30Gy or 20Gy [32] and with the advent of computed tomography, conformational imaging and now sophisticated dynamic imaging techniques used in planning, delivery of treatment is much more precise.

This de-escalation in fields and doses has been shown to permit greater protection of healthy breast tissue from the exposure to the radiation effect. Additionally, some evidence has suggested that this has been associated with significantly reduced breast cancer risk, although the risk still persists higher compared to general population [1, 9, 12, 13, 33]. Using a dosimetric risk modelling approach, Koh *et al*. [33] have demonstrated that the transition from 35Gy mantle radiotherapy to 35Gy IFRT has reduced the excess risk of breast cancer by 65% and the same risk is even more reduced by 40% when moving to 20Gy IFRT.

Prevention and surveillance modalities are also key in addressing the increased risk of secondary breast cancer. The evidence for the efficacy of most prevention and screening interventions however is generally extrapolated from other high-risk groups particularly BRCA1 or BRCA2 mutation carriers and has not specifically been measured in pretreated patient populations.

The role of prophylactic bilateral mastectomy in prevention is yet to be defined in this group. A randomised placebo-controlled phase IIB trial is underway to assess whether low-dose Tamoxifen for 2 years may reduce the risk of radiation-induced breast cancer in patients who have received thoracic irradiation (NCT01196936) [34]. However, this chemoprevention will not be helpful in preventing hormone receptor-negative breast cancers [35], which are the most frequent histological subtype that develop in HL survivors according to some reports [2, 36].

Generally, the primary aim of breast cancer screening is to reduce breast cancer mortality, and this should be assessed in randomised controlled trials. However, no such study has been unfortunately conducted among HL survivors, and the only data available so far that show the impact on mortality for this group of patients are provided by a mathematical simulation study published by Hodgson *et al*. [37]. This model has predicted for the first time that early screening (at 25 years) will reduce breast cancer mortality among HL survivors treated with mediastinal radiotherapy. More specifically, early detection with magnetic resonance imaging (MRI) over a 24-year period will prevent 13.92 to 14.35 deaths from breast cancer.

Data from the UK screening program has shown that most second breast cancers were detected at a pre-invasive or early stage and were without lymph node involvement [5], from which it would be reasonable to extrapolate that screening will yield clinical benefit for these patients but this has yet to be confirmed in terms of mortality outcomes.

The International Late Effects of Childhood Cancer Guideline Harmonization Group has proposed annual breast surveillance for HL survivors, who have received ≥20Gy thoracic radiotherapy before the age of 30 years [3]. Surveillance should start at 25 or at least 8 years after irradiation for at least up to 50 years, and should include mammography, MRI or both [3]. The ideal imaging modality is still controversial. Mammography appears to be able to detect most secondary breast cancers even in young women with dense breasts [6, 14]. Nonetheless, it exposes the body to additional radiation doses. Interestingly, combining breast MRI to mammography has showed increased sensitivity from 68 % to 94 % [38], but this is at the expense of higher costs, as well as further stress linked to the increased number of false-positive results, leading to unnecessary exams and biopsies.

Despite the international efforts to establish surveillance recommendations for HL survivors, a great proportion including our patient did not even know about their increased risk of developing secondary breast cancer and had never undergone neither self-breast exam nor mammography [6–8]. Thus, better awareness of clinicians about these risks can help with communication to these women about secondary breast cancer risk and should contribute to improved risk management.

## Conclusion

The de-escalation of HL treatments that has occurred over the years, with the limitation and more precise delivery of irradiated disease fields and the reduction of radiation doses, has paved the way for what we hope will be a significantly reduced risk of second breast cancer in this group of patients. Yet, there is still much to be done in raising awareness among survivors for their increased breast cancer risk.

The need to determine what constitutes adequate breast screening in these patients is also important, but studies with a high evidence level are needed to better evaluate benefits and limitations.

Finally, in view of the increased risk of cardiotoxicity and in the absence of a consensus for these survivors, individualised and concerted management remains the best option for the treatment of secondary breast cancer after HL.

## Abbreviations

EBRT: external beam radiation therapy; ELIOT: intraoperative radiotherapy with electrons; G: grade; HER2: human epidermal growth factor receptor 2; HL: Hodgkin lymphoma; IFRT: involved field radiotherapy; MRI: magnetic resonance imaging; mSBR: Scarf-Bloom-Richardson classification modified by Elston and Elliss; NA: not available; PBB: partial breast brachytherapy; PBI: partial breast irradiation; WBRT: whole breast radiotherapy.

## Competing interests

The authors declare that they have no competing interests.

## Authors’ contributions

JB designed the study, performed the literature review, drafted the manuscript and carried out the collection and assembly of data. MM analysed and interpreted the patient data regarding her oncological features and contributed to the revision of the manuscript. AL is responsible for the clinical management. All authors read and approved the final manuscript.

## Figures and Tables

**Table 1.** Published studies reporting conservative treatment among breast cancer patients previously treated for HL.

Study	Re-irradiation technique	Dose in Gy	Median follow-up in mo (range)	Toxicity events	Locoregional recurrence rate (%)	Cosmetic result
Wolden *et al*. [14] n = 2	WBRT with tangential fields	45.6, 46	72, 168^a^	Severe soft tissue necrosis	0	NA
Aref *et al*. [22] n = 2	WBRT	46, 48	27, 32^a^	No	0	Good to excellent
Deutsch *et al*. [18] n = 11	WBRT	50–51	46 (1–74)	No	0	Good to excellent
Cutuli *et al*. [23] n = 32	WBRT	NA	40 (10–192)	No	12.5	NA
Intra *et al*. [17] n = 6	ELIOT	17, 21	30.8^b^ (17–53)	No	8	Very good
Sana *et al*. [24] n = 33	EBRT, ELIOT	NA	41 (1–101)	NA	<5^d^	NA
El-Din *et al*. [19] n = 1	3D conformal PBI	50	27^a^	Moderate erythema	0	Excellent
Chadha *et al*. [25] n = 5	PBB	45	(5–67)	No	0	Excellent
Nguyen *et al*. [21] n = 2	WBRT	45, 50	43^c^ (6–127)	G1^c^	0	NA
Intra *et al*.^c^ [26] n = 35	ELIOT	17, 18, 21	52 (6–132)	G1, G2 subcutaneous tissue	9	Very good
Cutuli *et al*. [27] n = 56	WBRT	NA	50	No	13.7	NA
Haberer *et al*. [20] n = 30	WBRT	39.6–52.2	84 (4.8-384)	NA	7^d^	NA
Chadha *et al*. [28] n = 8	PBB	45^e^^,^ ^f^	73^f^	G1 skin pigmentation + G3 fibrosis^f^	1^d^	Excellent
Terenziani *et al*. [29] n = 29	NA	28–60	99	No	NA	NA
Burt *et al*. [30] n = 46	NA	NA	57 (0–299)	NA	NA	NA

a Follow-up in months;

b Average follow-up;

c Data for HL+NHL;

d Number of locoregional recurrences;

e Median dose;

f Data for patients with prior HL+ prior history of breast cancer.

**Abbreviations: EBRT:** External beam radiation therapy; **ELIOT:** Intraoperative radiotherapy with electrons; **G:** Grade; **NA:** Not available; **PBB:** Partial breast brachytherapy; **PBI:** Partial breast irradiation; **WBRT:** Whole breast radiotherapy.
